# Microsaccades mediate perceptual alternations in Monet’s “Impression, sunrise”

**DOI:** 10.1038/s41598-021-82222-3

**Published:** 2021-02-11

**Authors:** Robert G. Alexander, Ashwin Venkatakrishnan, Jordi Chanovas, Stephen L. Macknik, Susana Martinez-Conde

**Affiliations:** 1 Department of Ophthalmology, SUNY Downstate Health Sciences University, Brooklyn, NY USA; 2 Graduate Program in Neural and Behavioral Science, SUNY Downstate Health Sciences University, Brooklyn, NY USA

**Keywords:** Human behaviour, Perception

## Abstract

Troxler fading, the perceptual disappearance of stationary images upon sustained fixation, is common for objects with equivalent luminance to that of the background. Previous work showed that variations in microsaccadic rates underlie the perceptual vanishing and intensification of simple stimuli, such as Gabor patches. Here, we demonstrate that microsaccade dynamics also contribute to Troxler fading and intensification during the viewing of representational art. Participants fixated a small spot while viewing either a Gabor patch on a blank background, or Monet’s painting “Impression, Sunrise.” They continuously reported, via button press/release, whether the Gabor patch, or the sun in Monet’s painting, was fading versus intensifying, while their eye movements were recorded with high precision. Microsaccade rates peaked before reports of increased visibility, and dropped before reports of decreased visibility or fading, both when viewing Gabor patches and Monet’s sun. These results reveal that the relationship between microsaccade production and the reversal and prevention of Troxler fading applies not only to the viewing of contrived stimuli, but also to the observation of “Impression, Sunrise.” Whether or not perceptual fading was consciously intended by Monet, our findings indicate that observers’ oculomotor dynamics are a contributor to the cornerstone of Impressionism.

## Introduction

**Figure 1:**
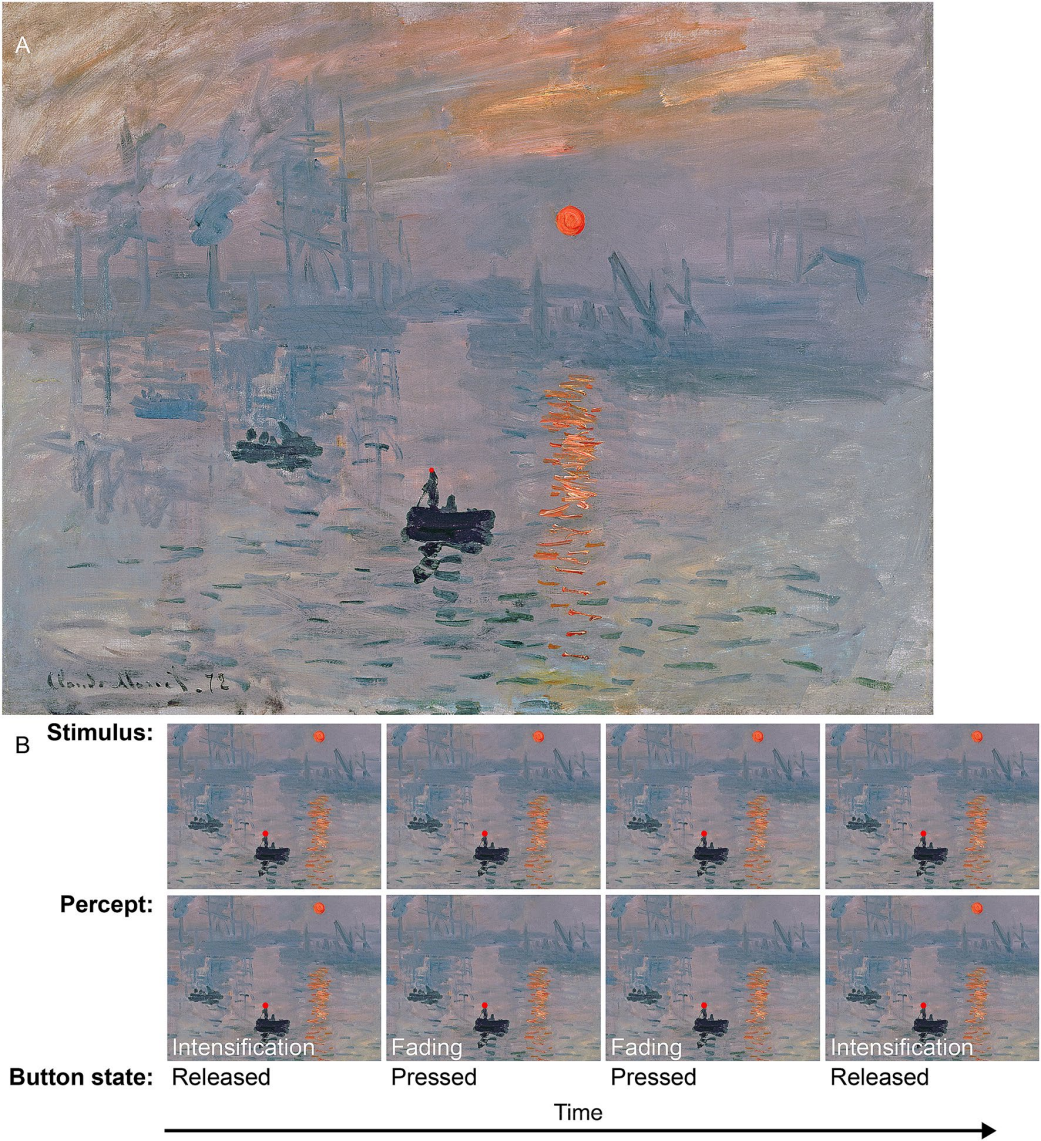
Experimental design. (**A**) Monet’s “Impression, soleil levant,” presented at its original orientation, with the addition of a red spot on the hat of the person standing in the boat. This red spot served as a fixation target during the experiment. To experience the perceptual fading of the sun, look at the red spot and hold your gaze still for about a minute while covertly focusing your attention on the sun. (**B**) Epochs from a Monet trial. The physical stimulus is shown in the top row, the participant’s perception of the stimulus in the second row, and the participant’s report in the third row. The perceptual experience is consistent with Troxler fading^1^: the sun disappears perceptually, and fills-in with the colour of the surrounding background. The fixation spot is not to scale. Reproduction from Musee Marmottan Monet, Paris, France/Bridgeman Images.

“Impression, Sunrise,” created by the French painter Claude Monet in 1872, is credited with inspiring the name of the Impressionist movement. The painting depicts the harbor of Le Havre in France, as seen from Monet’s window (Fig. 1A). This was not Monet’s actual view of the scene—as he explained—but his “impression,” hence the title. Indeed, one way in which “Impression, Sunrise” departs from reality is that the rising sun appears substantially brighter than the adjacent sky. Whereas this is true in life (i.e. the sun’s luminance is actually greater than that of the sky), the viewer’s perception of Monet’s sun as more brilliant than its surroundings is an illusion. Grayscale renderings of “Impression, Sunrise” show that the sun has the same physical luminance as the background clouds. This equiluminant quality may be critical to the painting’s “eerie” lifelike appearance, in which the sun “seems to pulsate” against the sky^1^.

Over two decades ago, Safran and Landis^2^ noticed that staring for a few seconds at the head of the sailor standing in the boat in Monet’s painting causes the sun to seemingly fade away. The perceptual result is a sunless sky, as the region occupied by the vanished sun fills in with visual information from the surrounding background. Safran and Landis suggested that such fading—and subsequent filling-in—of Monet’s sun is due to the phenomenon known as Troxler fading. This hypothesis is supported by prior reports that Troxler fading, the perceptual disappearance of stationary images upon sustained fixation, is quite common for peripheral objects with equivalent luminance to that of the background^3–6^. However, no previous studies have quantified the temporal dynamics of perceptual fading in “Impression, Sunrise,” or determined their potential relationship to ongoing fixational eye movements.

Here, we set out to determine if changes in the production of microsaccades—small, involuntary eye movements occurring a few times a second during fixation—drive the perceptual disappearance (and eventual reappearance) of the sun in Monet’s painting. Previous studies have shown that variations in microsaccadic rates underlie the perceptual vanishing and intensification of simple, contrived visual stimuli (i.e., Gabor patches), presented in the context of Troxler fading tasks^7–11^. Were a similar relationship between oculomotor and perceptual dynamics to be found for “Impression, Sunrise,” it would support the proposal that the illusory vanishing of Monet’s sun constitutes an instantiation of Troxler fading in the art domain. In addition, it would indicate the relevance of observers’ fixational eye movement dynamics to their experience of Monet’s masterpiece.

## Methods

### Participants

Twenty-two participants (12 males, 10 females; ages ranged from 15 to 54, with an average of 28.3 years), with normal or corrected-to-normal vision, participated in the experiment. Eighteen participants were naïve and were paid $15/session. All experiments were performed in accordance with relevant guidelines and regulations. Ethical clearance was approved by the SUNY Downstate Institutional Review Board (protocol number 690152) and the experiments were performed in accordance with the Declaration of Helsinki. Written informed consent was obtained from each participant. One additional participant was discarded, because their “Gabor Condition” data (which served as control for the “Monet Condition,” see below) failed to produce the known relationship between ocular events and perceptual fading dynamics^7,8,10^ that serves as the basis for comparison with the “Monet Condition” data in the present study (as detailed below).

### Experimental design

Participants rested their forehead and chin on the EyeLink 1000 head/chin support, ~ 72.0 cm away from a linearized video monitor (Barco Reference Calibrator V, 60 Hz refresh rate). The experiment consisted of 252 pseudorandomly-interleaved 30-s trials. After 30 s, all stimuli disappeared and the trial ended. To disregard the potential effect of the initial stimulus onset transient at the start of each trial, we conducted analyses only on data recorded after both the first second of the trial and the first button press (which indicated the first perceptual transition toward fading).

While fixating a small red spot (0.1° diameter), participants were asked to continuously report whether an unchanging stimulus (the sun in the Monet Condition, and a Gabor patch in the Gabor Condition) was faded/fading (button press) or intensified/intensifying (button release)^7–10^. The stimulus did not change physically, but it appeared to fade or intensify as a function of the observer’s fixation dynamics—see Fig. 1B. Naïve participants were not informed, before the experiment, that the only changes to the appearance of the stimulus would be illusory.

The experiment included three kinds of trials. Here we present data from the Monet Condition and the Gabor Condition only. The third type of trial consisted of images of an unrelated painting (72 trials total), which were used in a different project and not discussed further here.

### Monet condition

A reproduction of Monet’s “Impression, Sunrise” was presented on 36 trials, and a mirror-image of the same painting (horizontally-flipped, to lessen the effect of contrast adaptation across trials) was presented on another 36 interleaved trials. Thus, there were 72 “Monet” trials total, in which participants looked at a fixation target (a red spot of 0.1° diameter, placed on the hat of the standing fisherman), and simultaneously reported, at all times, whether the depicted sun was in the process of fading or intensifying (Fig. 1). The painting was centered on the monitor, in both its original orientation and as a horizontally-flipped mirror-image. As a result, the sun appeared on either the left or the right side of fixation. The painting’s digital reproduction used here was directly acquired from the Muséeum Marmottan Monet (which holds the original painting in their collection). This digital image had a resolution of 8241 × 6312, which we then down sampled to 1468 × 1125 to achieve a distance of 9° from the fixation target to the center of the sun (for the purpose of data comparisons between the Monet Condition and the Gabor Condition).

### Gabor condition

The Gabor Condition served as a control condition against which to compare the results from the Monet Condition. Thus, the Gabor trials were designed to reproduce the visual stimuli presented in prior research showing a causal relationship between changes in microsaccade dynamics and perceptual alternations during Troxler fading^7–11^. Following from this, we presented a two-lobe Gabor patch with peak-to-trough width of 2.5° (with axis parallel to the carrier grating). The Gaussian SDs were x = 1.5° and y = 1°, with a sinusoidal grating having a period of 5° and phase of 0. The Gabor had a maximum contrast of 40% from peak-to-trough and the same average luminance (50%) as the background. The position of the Gabor varied randomly across trials at one of eight points of the compass (0°, 45°, 90°, …, 315°) to control for possible contrast adaptation effects across trials. The orientation of the Gabor also varied randomly between 0° and 360° (35 different orientations varying in steps of 10° from 0° to 360°) in each trial to control for orientation adaptation effects. As on Monet trials, the distance from the fixation target (a red spot of 0.1° diameter placed on the center of the screen) to the center of the fading stimulus (i.e. the Gabor patch) was fixed at 9°. This condition included 108 trials.

### Eye movement analysis

**Figure 2:**
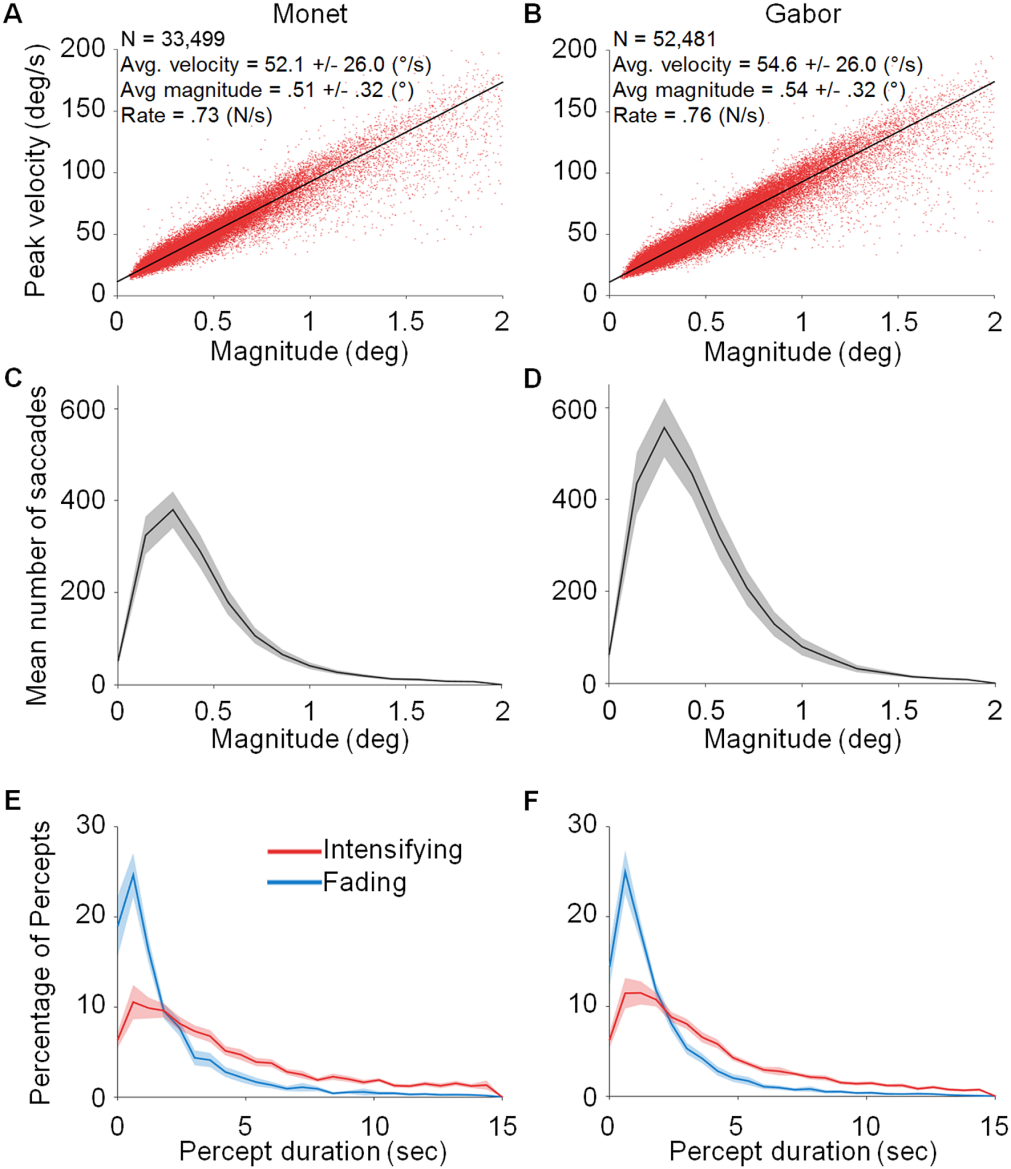
Microsaccade characterization and perceptual reports. (**A,B**) Peak velocity-magnitude relationship for all (micro)saccades with magnitudes < 2°. (**C,D**) Distribution of (micro)saccade magnitudes for the participant average. (**E,F**) Distribution of intensifying (red) and fading (blue) periods during the Monet and Gabor conditions, as indicated by the participants’ report. Shading indicates the SEM across participants. N = 22 participants for each plot.

**Figure 3:**
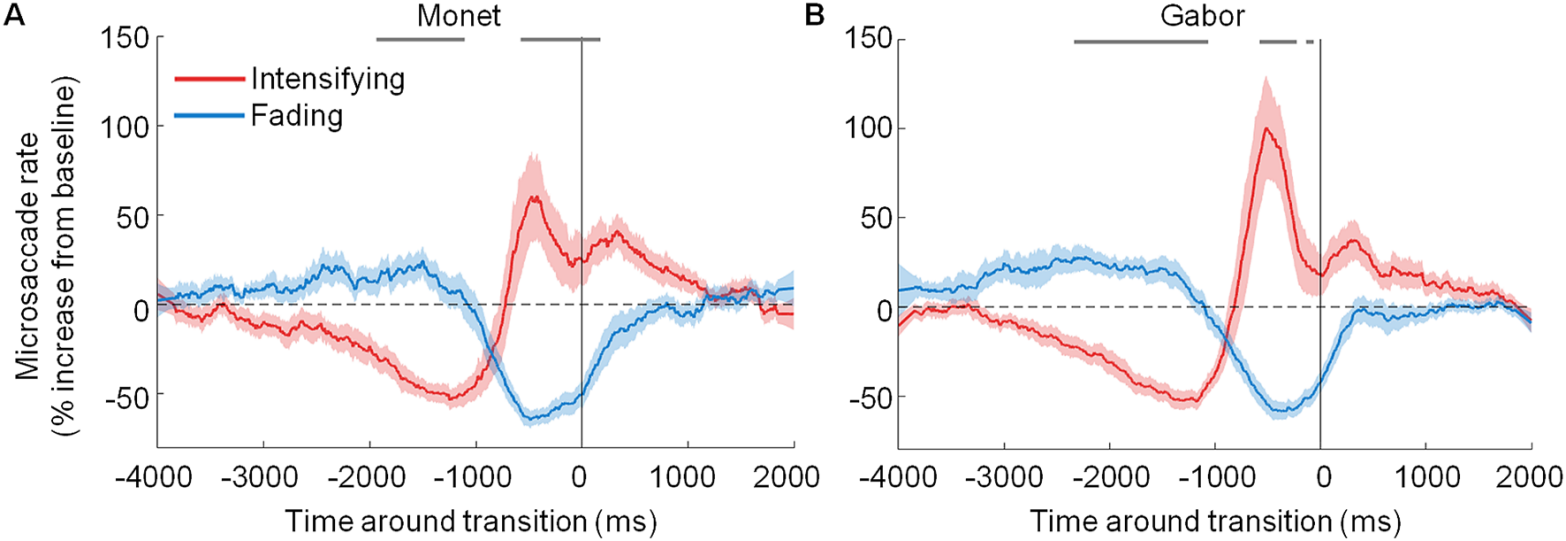
Microsaccade dynamics before transitions toward perceptual intensification versus fading. Average probability of microsaccades before transitions toward perceptual intensification (red) versus fading (blue) for (**A**) Monet’s sun and (**B**) a Gabor patch. The solid vertical line indicates the reported transitions (time = 0). The grey dashes along the top of the plots indicate the bins where microsaccade rates before transitions to intensification were significantly higher than microsaccade rates before transitions to fading (two-tailed paired *t* tests with Bonferroni correction, bin size = 20 ms, *p* < 0.05). Red and blue shading indicates the SEM across subjects (N = 22). Because trials from the two conditions were interleaved, both panels show data from the same participants, collected during the same sessions.

Eye position was acquired noninvasively with a fast video-based eye tracker at 500 Hz (EyeLink 1000, SR Research). We recorded eye movements simultaneously in both eyes, identifying and removing blink periods as portions of the raw data where pupil information was missing. We also removed portions of data where very fast decreases and increases in pupil area occurred (> 50 units per sample; such periods might indicate semi-blinks where the pupil is never fully occluded)^12,13^. In addition, we removed 200 ms of raw data before and after each blink/semi-blink to eliminate the initial and final parts where the pupil was still partially occluded^12^. Saccades were identified with a modified version of the algorithm developed by Engbert and Kliegl et al.^14–18^, with λ = 6 (used to obtain the velocity threshold) and a minimum saccadic duration of 6 ms. To reduce the amount of potential noise, we considered only binocular saccades, that is, saccades with a minimum overlap of one data sample in both eyes^15–18^. Moreover, we imposed a minimum intersaccadic interval of 20 ms so that potential overshoot corrections might not be categorized as new saccades^19^. To calculate microsaccade properties such as magnitude and peak velocity, we averaged the values for the right and left eyes. Figure 2 shows the peak velocity-magnitude relationship (Fig. 2A,B) and the magnitude distribution (Fig. 2C,D) for (micro)saccades with magnitudes up to 2°). All subsequent analyses (Fig. 3) concern microsaccades only, that is, saccades with magnitudes < 1°. (Saccadic magnitude thresholds of 1.5°, 2°, and 3° produced comparable results; see Supplementary Fig. 1).

### Microsaccade correlations with reported transitions

Let *X*_*M*_ and *X*_*R*_ be the stochastic processes representing the onsets of microsaccade, and intensification report (R). For example, if *S*_*1*_, *S*_*2*_, …, *S*_*k*_ are the start times of all the microsaccades for a given subject, then *X*_*M*_ for that subject will be given by *X*_*M*_(*t*) = 1 if *t* = *S*_*i*_ for some 1 ≤ *i* ≤ *k* and *X*_*M*_(t) = 0 otherwise; similarly for intensification reports.

We obtained correlations of microsaccades with reports of intensification for each subject, using $\xi$_*MR*_(t) $={\sum }_{m=-\infty }^{n=\infty }X$_m_(n + *t*)*X*_*R*_(*n*) and then converting it to a rate (similarly for transitions to fading)^8^. For each participant, correlations were smoothed using a Savitsky–Golay filter of order 1 and a window size of 151 ms^7^. Average correlations are the average of the smoothed correlations (Fig. 3).

### Microsaccade correlation baselines

For each condition (e.g. Monet or Gabor), we defined the microsaccade rate baseline as the rate of microsaccades produced far from the changes in visibility that took place during those trials. Thus, we calculated these microsaccadic rates using data 700 ms away from all reported transitions (i.e. perceptual intensification or fading) in both directions of time. The microsaccades produced during this period are independent of the perceptual transitions, as they occurred outside of subjects’ reaction times window in both directions of time^8,9^.

### Statistical methods

To test whether microsaccade rates before transitions to intensification were significantly higher than those before transitions to fading, we performed two-tailed paired t-tests in each bin (bin size = 20 ms), using Bonferroni correction to account for multiple comparisons (Fig. 3). All other statistical tests were likewise two-tailed paired t-tests. Significance levels were set to α = 0.01 throughout.

## Results

Participants fixated a small spot while viewing either a Gabor patch on a blank background, or Monet’s painting “Impression, Sunrise” (Fig. 1A). They continuously reported, via button press/release, whether the Gabor patch, or the sun in Monet’s painting, was fading versus intensifying. Their eye movements were recorded simultaneously with high precision (for details, see Materials and Methods). Figure 1B describes a typical epoch during a trial.

As with other bistable stimuli paradigms^7,20–23^, participants reported that their perception oscillated between two alternating states (faded/fading vs. visible/intensifying), both for the Gabor patch^7–11^ and for Monet’s sun^1^, even though neither stimulus changed physically over the course of a trial.

Both the Gabor and the Monet sun stimuli faded for a substantial amount of time, without a significant difference in percept duration (for either faded or visible percepts) between the Gabor and Monet conditions (*t*(42) = − 1.91, *p* = 0.06, 95% CI [− 4.08, 0.11] for intensification; *t*(42) = − 0.19, *p* = 0.85, 95% CI [-1.05, 0.87] for fading, see Fig. 2E,F).

Oculomotor behavior was also equivalent across the Gabor and the Monet conditions. For example, the microsaccade main sequences (peak velocity-magnitude relationships) were comparable for both types of trials (*t*(42) = 0.15, p = 0.88, 95% CI [− 4.83, 5.6], see Fig. 2A,B), and so were the microsaccade magnitude distributions, *t*(42) = − 0.42, *p* = 0.67, 95% CI [− 0.16, 0.1]; see Fig. 2C,D). See Table 1 for additional statistics regarding the participants’ oculomotor dynamics and perceptual reports.

**Table 1:** Average microsaccade and perceptual parameters.

	Monet condition	Gabor condition
Number of trials	72	108
Number of microsaccades	1399 ± 127 per subject (19.43 ± 1.76 per trial)	2165 ± 192 per subject (20.05 ± 1.78 per trial)
Microsaccade rate (*N*/s)	0.67 ± 0.06	0.69 ± 0.06
Microsaccade magnitude (°)	0.44 ± 0.02	0.47 ± 0.02
Microsaccade duration (ms)	14.33 ± 0.65	15.18 ± 0.69
Microsaccade peak velocity (°/s)	47.09 ± 1.89	49.37 ± 2.08
Number of transitions to fading (*N*)	215.27 ± 28.01 per subject (2.99 ± 0.35 per trial)	405.82 ± 39.28 per subject (3.76 ± 0.33 per trial)
Duration of perceived fading periods subsequent to the first 1 s of the trial (s)	6.95 ± 0.87 total per trial	8.32 ± 0.83 total per trial
Duration of first fading period after stimulus onset (s)	3.04 ± 0.61	2.76 ± 0.41
Time spent in fading periods (% of trial)	23.96 ± 3.00	28.67 ± 2.87
Number of transitions to intensifying (*N*)	192.23 ± 26.96 per subject (2.67 ± 0.34 per trial)	368.54 ± 37.81 per subject (3.41 ± 0.35 per trial)
Duration of perceived intensifying periods subsequent to the first 1 s of the trial (s)	9.12 ± 0.62 total per trial	11.27 ± 0.43 total per trial
Time spent in intensifying periods (% of trial)	31.44 ± 2.15	38.82 ± 1.47

Number of microsaccades, microsaccade rate, magnitude, duration, and peak velocity, were calculated for saccadic movements with a magnitude ≤ 1°.

Next, we examined the timing of microsaccades with respect to that of perceptual alternations, by locking changes in microsaccade rates to changes in perceptual transition reports. This analysis revealed a clear relationship between microsaccade production and fading/intensification dynamics. Specifically, we found that increased microsaccade rates were related to perceptual intensification/visibility. Conversely, decreased microsaccade rates were related to perceptual fading (Fig. 3 and Supplementary Figs. 1 and 3). Importantly, the relationship between microsaccade production and the perceptual intensification/fading of Monet’s sun mirrored such a relationship in the case of the Gabor patch—which itself replicated previous findings connecting microsaccade production to the reversal of Troxler fading^7–11^.

Our combined results provide the first quantification of eye movement dynamics as related to the perception of visibility in a critical element of Monet’s masterpiece. They moreover suggest that the vibrant, pulsating quality of Monet’s sun^1^ results from the viewer’s own oculomotor behavior.

## Discussion

In this study, we show that changes in microsaccade production underlie the intermittent vanishing and reappearance of the sun in Claude Monet’s “Impression, Sunrise,” thereby indicating that such perceptual alternations are an instantiation of Troxler fading in this iconic masterpiece.

Prior work has linked Troxler fading to neural adaptation to unchanging stimuli^24–26^. Abrupt retinal motion from microsaccades (and other transient oculomotor events, such as larger saccades and blinks) has been shown to counteract such adaptation and consequently restore the visibility of previously faded visual objects^7–11^. However, no prior study had demonstrated the impact of this perceptual phenomenon in a viewer’s experience of visual art, especially in connection to the observer’s own gaze behaviour. The effects of changing microsaccade dynamics on Troxler fading have been previously shown with Gabor patches, well-controlled visual stimuli that are frequently used in psychophysical experiments^7–11^. Here, we asked if microsaccade production had comparable effects on the visibility of both Gabor patches and the sun in Monet’s painting. Thus, our experimental design included a number of Gabor trials, in which the stimuli characteristics precisely replicated those used in our earlier research^8,10^. Consequently, we were able to correlate microsaccades to perceptual reports for both Gabor patches and Monet’s sun, and compare the results across the two types of trials.

Time-locked analyses of microsaccades and perceptual reports (button presses and releases) revealed several key findings. First, in agreement with previous studies^8–11^, we found that microsaccade rates decreased before reports of perceptual fading and increased before reports of perceptual intensification, showing that microsaccades both prevent and counteract the incidence of Troxler fading in Gabor patches. Second, we extended the above findings to a more naturalistic context: the viewing of artwork. We demonstrated that the relationship between microsaccade production and the reversal and prevention of Troxler fading applies not only to the viewing of contrived stimuli such as Gabor patches, but also to the observation of Monet’s masterpiece. Oculomotor and perceptual dynamics, and the relationship between them, were quantitatively equivalent for Gabor and Monet trials, including but not limited to the duration of fading and intensification periods, the slope of the (micro)saccadic peak velocity-magnitude relationship, and the temporal correlations between microsaccade onsets and perceptual reports of fading and intensification. We note that the relationship between microsaccade dynamics and target visibility found here is also likely to apply to other transient oculomotor events (including larger saccades and blinks), as previously reported for the perception of Gabor patches during Troxler fading^8,9^, as well as that of illusory motion from static repetitive patterns^27^.

We might speculate as to whether Monet—intentionally or intuitively—controlled the composition so that the sun faded when viewers sustained their gaze upon a specific focal point of the painting—such as the sailor. It is widely accepted that artists emphasize and exploit the perceptual skills of observers through both creative intuition and artistic skill^28^. Thus, Monet may have adjusted the visual qualities of his painting to produce a depiction of the sun that is prone to fading (specifically, by minimizing the luminance contrast between the sun and its immediate surroundings). In that case, Monet could have intended the perceptual fading of the sun, making specific choices about the visual organization in “Impression, Sunrise” to influence the viewers’ experience of the art. Alternatively, Monet may have chosen the properties of the depicted sun for other purposes, in a way that coincidentally facilitates fading.

Whether or not perceptual fading was calculated by Monet, the qualities of depicted objects hold the potential to alter viewers’ experience of the artwork. Our results support the proposal that Troxler fading drives the illusory vanishing of the sun in Monet’s “Impression, Sunrise,” and suggest that observers’ oculomotor dynamics are a meaningful contributor to their perception of this cornerstone of Impressionism.

## Supplementary Information

Supplementary Information
