# Supplementary material for: Microsaccades mediate perceptual alternations in Monet’s “Impression, sunrise”
Source: Sci Rep. 2021 Feb 11;11:3612. doi: 10.1038/s41598-021-82222-3 (PMC7878487; doi:10.1038/s41598-021-82222-3)
Supplement: Supplementary file 1 — Supplementary Information [file 41598_2021_82222_MOESM1_ESM.docx]

Microsaccade Dynamics Mediate Perceptual Alternations in Monet’s “Impression, Sunrise”

Robert G. Alexander^1^

Ashwin Venkatakrishnan^1^

Jordi Chanovas^1, 2^

Stephen L. Macknik^1^

*Susana Martinez-Conde^1^

^1^Department of Ophthalmology, SUNY Downstate Health Sciences University, Brooklyn, NY USA

^2^Graduate Program in Neural and Behavioral Science, SUNY Downstate Health Sciences University, Brooklyn, NY USA

*Corresponding author: S.M.-C. (email: smart@neuralcorrelate.com). SUNY Downstate Medical Center, 450 Clarkson Ave MSC 58, Brooklyn, NY 11203, USA

# Supplemental Materials

**Supplemental Figure 1: Dynamics of (micro)saccades of different magnitudes before perceptual transitions.** In **Figure 3**, microsaccades of magnitudes <1° are correlated to reported perceptual transitions. Here we present equivalent analyses for (micro)saccades of magnitudes <1.5° (**A,B**), <2° (**C,D**), and <3° (**E,F**). In all cases, microsaccade rates increased shortly before transitions to perceptual intensification and decreased before transitions to perceptual fading, both for Gabor patches and for Monet’s sun. The solid vertical line indicates the reported transitions (time=0). The grey dashes along the top of the plots indicate the bins where microsaccade rates before transitions to intensification were significantly higher than microsaccade rates before transitions to fading (two-tailed paired *t* tests with Bonferroni correction, bin size=20 ms, p value<0.05). Red and blue shading indicates the SEM across subjects (N=22).

**Supplemental Figure 2: Fixation maps from the Monet (A) and Gabor (B) conditions.** Black dots denote the Gabor/sun stimulus location in each experimental condition. Dashed lines in **B** and **D** indicate the horizontal and vertical meridians; the dashed circles indicate all x,y coordinates that are equidistant with the Gabor stimulus. Panels **C** and **D** show the same data as in **A** and **B**, but collapsed across stimuli locations. Specifically, the x,y coordinates of the fixation data for the mirror-image Monet trials were horizontally-flipped, such that the location of the fixation target and all details of the painting matched those in the original orientation. Fixation data from the Gabor condition were rotated so as to align all trials, with the Gabor located at 0°. After spatially aligning each trial, we assessed eye position variability using Bivariate contour ellipses (BCEAs), which represent the regions over which eye positions were found 68% of the time. BCEAs are visualized in **C** and **D** as green ellipses, with the center of each ellipse indicated by a green +. The BCEA was significantly higher for Monet trials (25.27 deg^2^ visual angle) than for Gabor trials (45.16 deg^2^), *t*(42)=-3.465, *p*<.01. This difference likely resulted from the richer background in Monet’s painting (i.e. compared to the blank background in the Gabor condition) prompting participants to briefly gaze away from the fixation target to scrutinize details such as the contours of other boats, or Monet’s signature.

**Supplemental Figure 3: Dynamics of microsaccades of different directions before perceptual transitions.** Microsaccades with directions towards the Gabor/sun stimulus (±90°) for the Monet (**A**) and Gabor conditions (**C**) are considered separately from microsaccades with directions >90° away from the Gabor/sun stimulus for the Monet (**B**) and Gabor conditions (**D**). The data indicate that microsaccades heading both toward and away from the Gabor/sun stimuli contributed to the perceptual alternations observed. The grey dashes along the top of the plots indicate the bins where microsaccade rates before transitions to intensification were significantly higher than microsaccade rates before transitions to fading (two-tailed paired *t* tests with Bonferroni correction, bin size=20 ms, p value<0.05). Red and blue shading indicates the SEM across subjects (N=22).
